# Microbiome Analysis of Area in Proximity to White Spot Lesions Reveals More Harmful Plant Pathogens in Maize

**DOI:** 10.3390/biom15020252

**Published:** 2025-02-09

**Authors:** Sauban Musa Jibril, Yanping Hu, Kexin Yang, Jie Wu, Chengyun Li, Yi Wang

**Affiliations:** 1 State Key Laboratory for Conservation and Utilization of Bio-Resources in Yunnan, Yunnan Agricultural University, Kunming 650201, China; saubanzango@gmail.com (S.M.J.); huyanping@zohomail.cn (Y.H.); 18468006252@163.com (K.Y.); 15348635910@163.com (J.W.); 2 Yunnan-CABI Joint Laboratory for Integrated Prevention and Control of Transboundary Pests, Yunnan Agricultural University, Kunming 650201, China

**Keywords:** maize white spot, microbiome, diseased, spot proximity, healthy leaf

## Abstract

Plant microbiomes play a major role in plant health, growth, and development, enhancing resistance to pathogen invasion. However, despite the extensive research on the phyllosphere microbiome, it remains unclear how the microbiome of leaves in proximity to diseased leaves responds to pathogen invasion. We investigate the response of the maize phyllosphere microbiome to maize white spot by assessing the microbiome dynamics associated with the white spot portion and the area in proximity using 16S and ITS high-throughput sequencing analysis. Our results showed that the bacterial diversities were higher in the diseased portion and area in proximity to the spot than those in healthy plants. At the same time, lower fungal diversity was recorded in the diseased portion compared to portions in proximity to it and healthy leaves. The spot portion had a significant influence on the microbial composition. The diseased portion, the area in proximity to it, and the healthy leaves were dominated by the bacterial genera *Sphingomonas, Delftia, Chryseobacterium, Stenotrophomonas, Methylobacterium-methylorubrum,* and *Bacteroides*. Still, the abundance of *Sphingomonas* decreased in the healthy leaves with a corresponding increase in *Stenotrophomonas*. Conversely, the fungal genus *Setophoma* dominated the diseased portion, while the fungal pathogens *Cladosporium, Alternaria,* and *Exserohilum* were highly abundant in the samples from the area in proximity to it. In addition, a co-occurrence network analysis revealed a complex fungal network in healthy leaves and those in proximity to leaves infected with white spot compared to the diseased portion. This study suggests that the area in proximity to the maize leaf infected with white spot disease is colonized by more harmful plant pathogenic fungi for disease progression.

## 1. Introduction

Plants harbor a complex immune system to fight off invaders and prevent diseases. Many studies have demonstrated the beneficial role of plant microbiomes in disease suppression [1,2,3]. The phyllosphere, consisting of the aerial parts of plants, dominated by the leaves, is the habitat of abundant and diverse nonpathogenic microorganisms [4]. The phyllosphere microbiome is indispensable for plant health, protecting against foliar pathogens, and maintaining the crop yield and quality of agricultural products [5,6]. Plants recruit beneficial microbes to enhance their ability to fight pathogens [7]. The phyllosphere microbiome suppresses pathogenic microorganisms through competition for nutrients and space, antibiosis, and stimulating systemic host responses [4] by triggering the plant immunity-based reassembly of the microbiome [8]. Changes in the phyllosphere microbial composition have been identified in response to pathogenic invasion by *Diaporthe citri* in citrus [9], the development of angular leaf spot in cucumber [10], and rust infection in Malus [7]. Similarly, the fungal pathogen *Zymoseptoria tritici* causes systemic-induced susceptibility and a reduction in the bacterial richness in wheat-susceptible cultivars [11] and in the microbial diversity and composition of *Didymella segeticola*-infected tobacco [12]. Maize (*Zea mays* L.) is one of the world’s most important food and industrial crops cultivated globally and has played an increasing and diverse role in global agri-food systems [13]. In 2020, the production was estimated at 1.05 million tons, with China and the USA being the top producers [14]. Maize suffers considerable losses from biotic and abiotic stresses, seriously impacting the crop yield. Among the biotic stresses, maize leaf spot disease, leaf blight, and maize white spot (MWS) are the most common and damaging foliar diseases posing a significant threat to maize cultivation in tropical and subtropical regions of the world by reducing the crop yield [15,16,17].

Maize white spot (MWS) disease is among maize’s most detrimental foliar diseases, causing considerable losses in maize yield [18]. The disease was reported in maize fields in some areas in the Sichuan, Yunnan, and Hubei provinces in southwestern China [19]. The disease symptoms appear as chlorotic water-soaked spots on leaves [20]. The causative agent of maize white spot disease has been a subject of controversy. Several studies have reported different pathogens to be responsible for the disease. The fungus *Phaeosphaeria maydis* was initially reported to cause leaf spots [21,22]. However, the bacterial pathogen *Pantoea ananatis* was also reported as a causal pathogen of MWS [23,24]. Fungal pathogens that are members of the *Bipolaris* species are also reported to cause maize leaf spot [25,26]. The pathogen was formerly referred to as *Helminthosporium,* which was further differentiated into the genera Exserohilum, Bipolaris, and Drechslera and are all common foliar corn pathogens [27,28]. Isolates of *Exserohilum rostratum* from plant sources and isolates of the genus *Bipolaris* are closely similar in phylogeny, morphological features, and pathogenicity [29]. *Exserohilum rostratum* was reported to cause maize leaf spot in corn across the world, including in the United States [27]. The leaf spot foliar symptoms exhibited by maize when infected with these species have been found to be similar under field conditions [26]. Another bacterial strain, *Epicoccum latusicolum*, was predominantly associated with diseased maize leaves showing typical symptoms of MWS disease [15]. Recently, *Diaporthe eres* was reported as a causal agent of maize white spot in Yunnan Province, China [30].

Assessing the diversity, dynamics, and composition of the phyllosphere microbial community is a critical step for understanding its impact on plant health and development [31]. However, despite extensive studies on the phyllosphere microbiome, little attention has been paid to the response of plant-associated microbial communities to the site of pathogen infection, the area in its proximity, and the healthy area. This study was conducted to investigate the effect of maize white spot on the maize leaf microbiome at the site of infection, the area in proximity to the leaf spot, and the healthy area. In addition, we analyzed a microbial co-occurrence network and functional profiles to generalize the response of the maize phyllosphere microbiome to determine whether the infection site has an influence on the interactions and functions of the leave microbiome.

## 2. Materials and Methods

### 2.1. Sample Collection

**Figure 1:**
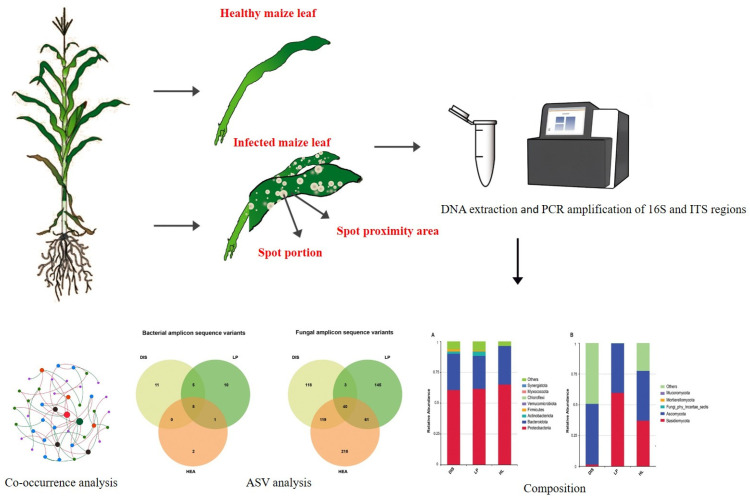
Overview of the experimental design and sampling site for this study.

Maize leaves exhibiting typical leaf spot symptoms were collected from Yunxian in Yunnan Province, China, in February 2024. The samples were categorized into three groups: diseased leaves (white spot portion), spot proximity (5 cm away from the lesion), and healthy leaves from the same plant to ensure consistency in microbial communities. The collected samples were placed in polythene bags, stored in a dry ice box, and transported to the laboratory for DNA extraction and analysis (Figure 1).

### 2.2. DNA Extraction and Amplification

Total genomic DNA was extracted using a PowerSoil DNA isolation kit (DNeasy PowerSoil Kit, QIAGEN, Hilden, Germany) according to the manufacturer’s instructions. The V4 region of the 16S gene was amplified with the primer set 515F (5′-GTGYCAGCMGCCGCGGTAA-3′) and 806R (5′-GGACTACNVGGGTWTCTAAT-3′) and the ITS2 was amplified using the primers set (5′-GCATCGATGAAGAACGCAGC-3’) and (5′-TCCTCCGCTTATTGATATGC-3′). All PCR reactions were carried out with 15 µL of Phusion^®^ High-Fidelity PCR Master Mix (New England Biolabs, Ipswich, MA, USA), 0.2 µM of forward and reverse primers, and about 10 ng template DNA. Thermal cycling consisted of initial denaturation at 98 °C for 1 min, followed by 30 cycles of denaturation at 98 °C for 10 s, annealing at 50 °C for 30 s, and elongation at 72 °C for 30 s and 72 °C for 5 min.

### 2.3. Library Sequencing and Processing

Paired-ends reads generated after sequencing were truncated by removing the barcode and primer, then merged using FLASH [32], and quality filtered using the fastp software [33]. Silva and UNITE databases were used to detect chimeric sequences in the 16S bacterial and ITS fungal data, and the sequences were filtered using VSEARCH V2.16.0 [34]. The non-chimeric sequences were denoised using DADA2 in QIIME2, and the amplicon sequence variants (ASV) were generated [35]. The taxonomic annotation of bacterial and fungal ASVs was against Silva and UNITE databases in QIIME2 [36,37]. The ASVs assigned to the chloroplast, mitochondria, unassigned, and host sequences were removed from the samples before further analyses. The High-throughput sequencing of 16S rRNA and ITS was conducted on the Illumina novaseq 6000 platform at Novogene Co., Ltd., Beijing, China.

### 2.4. Bioinformatics Analysis

QIIME 2 was used to calculate the alpha diversity indices (Chao 1, Shannon, and Simpson), which were visualized in R. For beta diversity, Principal Coordinate Analysis (PCoA) based on the unweighted and weighted UniFrac distance was performed to highlight differences in microbial communities among the spot portion, the area in proximity to it and healthy leaves. The statistical differences between the beta groups were tested using PERMANOVA with Adonis2 function in the R package vegan [38]. The relative abundance of the ten most abundant bacterial and fungal communities was assessed at both the phylum and genus levels. The Duncan test was employed to calculate differences among bacterial and fungal genera (*p* < 0.05). Intra-kingdom microbial network analysis was conducted using Sparse Correlations for Compositional data algorithm (SparCC) in R for ASVs at the genus level (*p* < 0.05 and correlation coefficient > 0.3) [39]. The networks were visualized in Gephi (0.9.2), and network properties (degree and betweenness centrality) were calculated and illustrated in Gephi. Hub taxa were defined as the top 5 nodes with high degree and closeness centrality. Functional prediction of bacterial communities was conducted using the Phylogenetic Investigation of Communities by Reconstruction of Unobserved States PICRUSt (V1.1.4) algorithm [40]. The Fungi Functional Guild (FunGuild) was used for the functional prediction of fungal communities. Constrained principal coordinated analysis (cPCoA) and the Shannon index were used to visualize the functional differences among the samples.

## 3. Results

### 3.1. Analysis of Sequence Amplicon Variants

**Figure 2:**
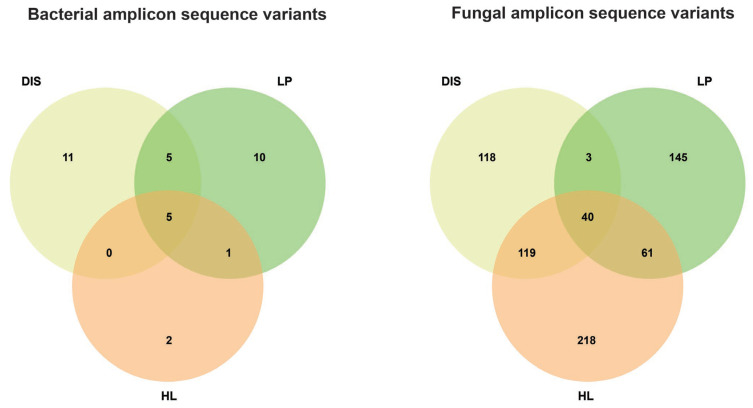
Distribution of bacterial (**left**) and fungal (**right**) amplicon sequence variants in diseased, disease proximity, and healthy leaves samples.

Analysis and distribution of amplicon sequence variants (ASVs) were conducted among the three samples. The maximum count of bacterial ASVs was recorded in diseased (DIS) and the disease proximal area (LP) compared to healthy leaves (HL). A total of 11, 10, and 2 specific ASVs were recovered in diseased, disease proximity, and healthy leaves samples, respectively, while 5 ASVs were shared among all the samples (Figure 2). For fungal communities, healthy areas in proximity to disease samples exhibited higher diversity and richness in ASVs than the diseased samples. The number of specific ASVs was significantly higher in the healthy sample (218) and in the disease proximity sample (145) than in the diseased sample (118), with 40 common ASVs shared among all the samples. These results suggest that maize white spot significantly affected both bacterial and fungal communities in the diseased portion and its proximity.

### 3.2. Effect of White Spot Location and Distance from the Spot on Beta Diversity

**Figure 3:**
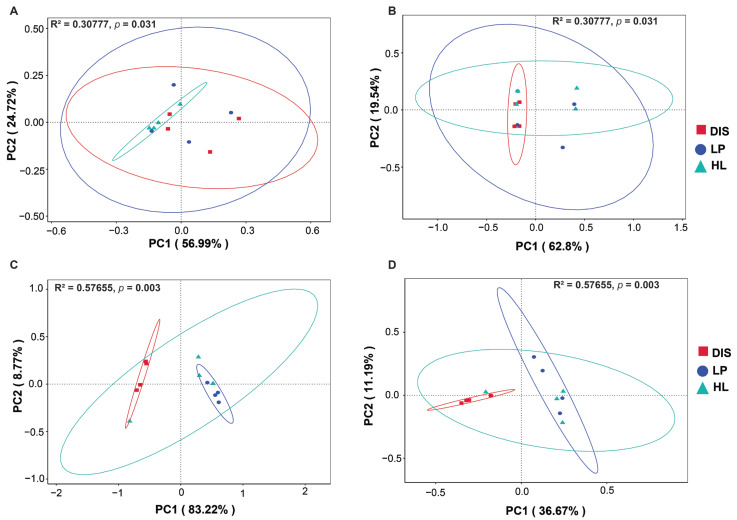
Principal coordinate analysis (PCoA) based on weighted and unweighted UniFrac displaying the beta diversity metrics of bacterial (**A**,**B**) and fungal (**C**,**D**) communities of maize leaf spot, spot proximity and healthy area. DIS, diseased, LP, disease proximity, HL healthy samples.

The impact of the nature of maize leaf samples (spot, spot proximity, and healthy) on bacterial and fungal β-diversity was assessed using Principal Coordinate Analysis (PCoA) based on both unweighted and weighted UniFrac (Figure 3). Both the weighted and unweighted PCoA analysis indicated that diseased samples were comparable to disease proximity and healthy samples. For bacteria, the variation explained by the two axes for both plots was 81.71% for weighted UniFrac and 82.34% for unweighted UniFrac (Figure 3A). For the fungal community, the variation explained by the two axes was 91.99% and 47.86%, respectively (Figure 3B). Permutational Multivariate Analysis of Variance (PERMANOVA) and pairwise comparison further indicated significant variation in the bacterial communities (R^2^ = 0.30777, *p* < 0.031) and fungal communities (R^2^ = 0.57655, *p* < 0.003). (Supplementary Tables S1 and S2). These results demonstrate that maize white spot lesions significantly influenced the diversity of the fungal community compared to the bacterial community.

### 3.3. Effects of White Spot Location and Distance from the Lesion on the Alpha Diversity

**Figure 4:**
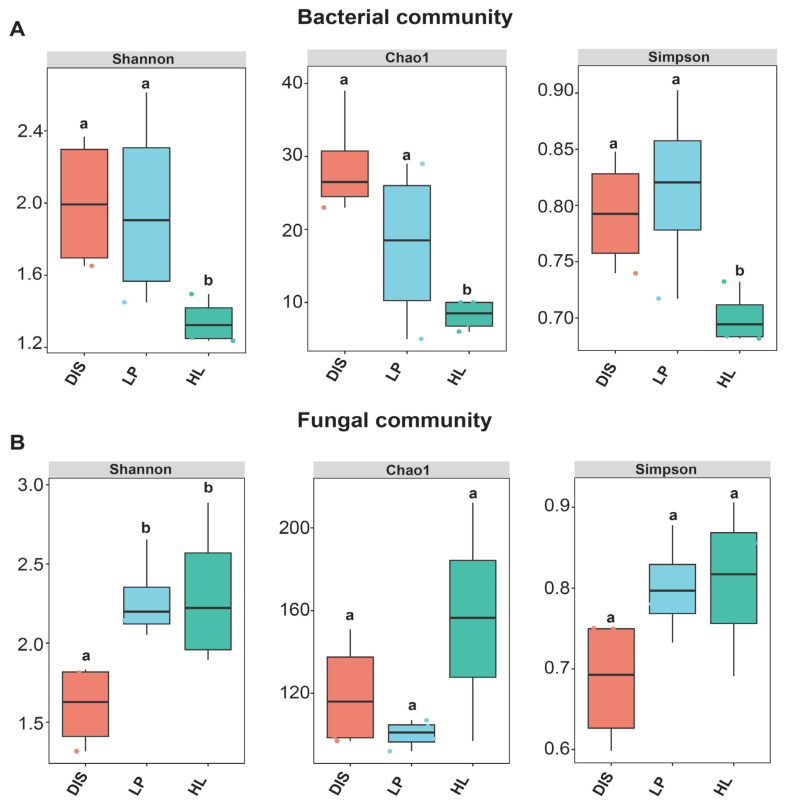
Boxplot of bacterial (**A**) and fungal (**B**) showing alpha diversity indexes in the leaf spot, spot proximity, and healthy maize leaf area. Different lowercase indicates significant differences (Kruskal–Wallis test *p* < 0.05).

The diversity among the white spot portion, its proximity, and healthy leaf was investigated. The results showed that the Shannon, Chao1, and Simpson indices were significantly higher in the maize leaf spot diseased area and its proximity compared to healthy leaves in the bacterial community (Figure 4A) (*p* < 0.05). This indicates that maize white spot increases the diversity of maize bacterial community with a similar effect observed in the spot proximity. In contrast, the fungal community displayed an opposite trend in α diversity. The Shannon and Simpson indices were greater in both the spot proximity and healthy leaves compared to the diseased portion, with spot proximity exhibiting the lowest Chao 1 index. However, no significant differences were found among Chao1 and Simpson indices of the fungal communities (Figure 4B) (*p* < 0.05). This suggests a considerable decline in the diversity of the maize leaf fungal community after invasion by the pathogen, indicating that the microbial community within the spot is also adversely affected.

### 3.4. Bacterial and Fungal Community Composition

**Figure 5:**
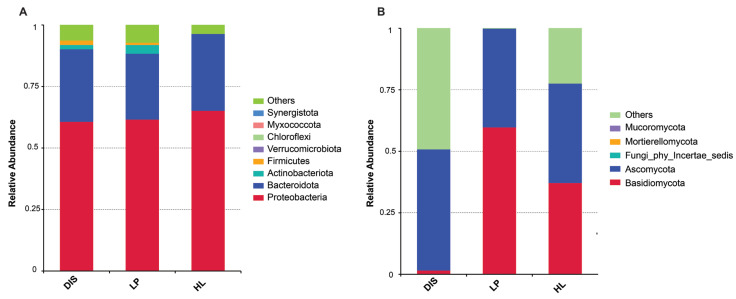
Maize microbial community composition among the white spot portion, its proximity, and healthy leaves samples. Relative abundance of the top 10 most abundant bacterial (**A**) and fungal (**B**) phyla.

The shift in the microbial community composition among maize white spot lesions, its proximity, and healthy samples was investigated, and the relative abundances of the top 10 bacterial and fungal taxa were visualized. The dominant bacterial phyla in the maize white spot portion and its proximity were Proteobacteria, Bacteroidota, Actinobacteriota, and Firmicutes, while the healthy samples were dominated by Proteobacteria and Bacteroidota (Figure 5A). For the fungal community, Ascomycota was the dominant phylum, with Basidiomycota in low abundance in the disease sample. Interestingly, the disease proximity and healthy leaves showed a considerable increase in Basidiomycota abundance (Figure 5B).

**Figure 6:**
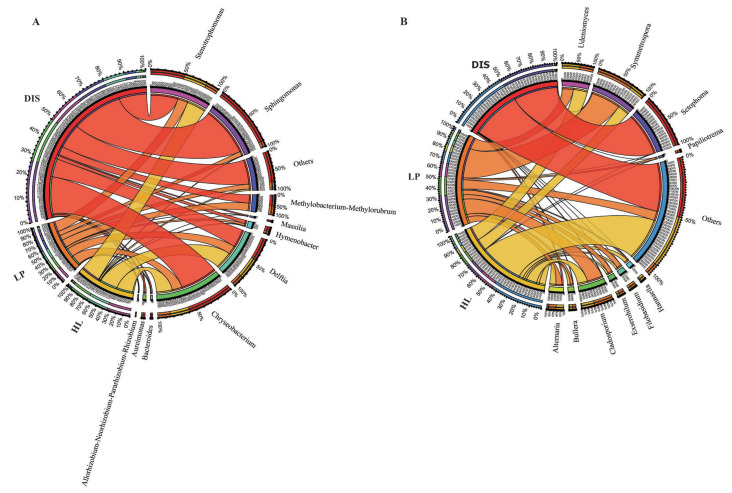
Chord diagram showing the relative abundance of the top 10 genus level. Relative abundance of the top ten bacterial (**A**) and fungal (**B**) genera.

At the genus level, the relative abundance of the top 10 bacterial and fungal genera was assessed to determine changes in their abundance in response to the maize white spot in the diseased portion, its proximity, and healthy leaves. The most abundant bacterial genera in the diseased portion and its proximity samples were *Sphingomonas, Delftia, Chryseobacterium, Stenotrophomonas, Methylobacterium-methylorubrum, Hymenobacter,* and *Bacteroides*. Notably, the relative abundance of *Sphingomonas* and *Chryseobacterium* decreased while the abundances of *Delftia, Stenotrophomonas, Methylobacterium-methylorubrum,* and *Hymenobacter* increased in the lesion proximity (LP). Genera *Delftia, Chryseobacterium,* and *Stenotrophomonas* were abundant in healthy leaves (HEA) (Figure 6A). Among the top ten genera, the abundance of *Symmetrospora was* significantly higher in healthy than in diseased and area proximity to the diseased (Duncan test, *p* < 0.05; Figure S1). Fungal genera *Setophoma, Symmetrospora,* and *Alternaria* were the most abundant in the diseased portion of the leaf. In the spot proximity, the fungal genera were dominated by *Symmetrospora, Udeniomyces, Cladosporium, Alternaria, Exserohilum, Papiliotrema Filobasidium* and *Bullera* (Figure 6B). The genus *Setophoma* was significantly higher in maize white spot portion compared to its proximity and healthy leaves, whereas genera such as *Symmetrospora, Cladosporium,* and *Filobasidium* exhibited opposite trends (Duncan test, *p* < 0.05; Figure S2). Analysis of maize pathogenic microbes revealed that fungal pathogens such as *Alternaria, Cladosporium, Phaeosphaeria, Exserophilum, Bipolaris, Botrytis,* and *Bullera* had higher abundances in the white spot proximity and healthy leave compared to the white spot portion (Figure S3).

### 3.5. Characteristics of Microbial Co-Occurrence Network

**Figure 7:**
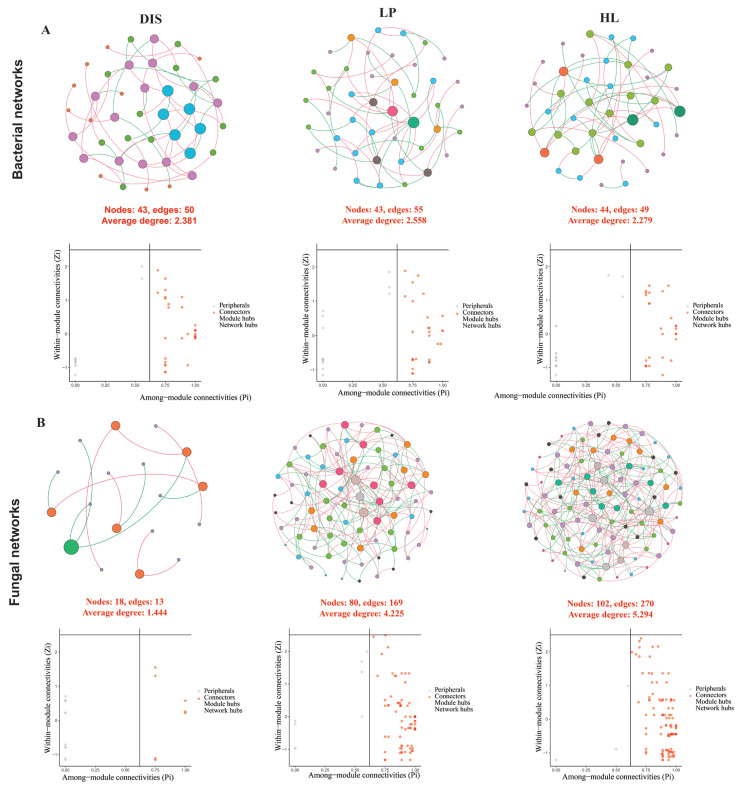
Bacterial and fungal microbial communities’ co-occurrence network analysis associated with the diseased, disease proximity and healthy samples in bacterial (**A**) and fungal (**B**) communities.

The bacterial and fungal microbial co-occurrence networks for the white spot portion, its proximity, and healthy leaves were constructed at the genus level. In the bacterial community network, the number of edges and average degree were higher in the spot portion and its proximity compared to the healthy samples. However, the healthy showed greater modularity and average path length, suggesting increased stability (Figure 7A). In contrast, the white spot portion and its proximity networks had the lowest number of nodes, edges, and average degree compared to the healthy leaf in the fungal network. The spot portion exhibited significantly fewer interactions with nodes, 18 edges, and a lower average degree, modularity, and average path length (Figure 7B). The number of nodes, edges, and average degree were significantly higher in the healthy network (Table 1). These findings suggest that maize white leaf spot may be the complexity of fungal interactions compared to bacteria.

Based on within-module (Z*_i_*) and among-module (P*_i_*) connectivity, networks can be categorized into four topological classes: peripherals, connector, module hub, and network hub. No module hubs and network hubs were detected in any of the networks. The topological role of the genera in each network was identified using P*i* and Z*_i_* and 12 genera were classified into peripherals and 30 assigned to connectors in the white spot portion of the bacterial network. The peripherals have 16 and 15 genera in the spot proximity and healthy networks, respectively, while connectors included 25 and 27, respectively (Table S3). The connector genera, which include *Rhodococcus, Pseudomonas, Ralstonia,* and *Faecalibacterium,* were present in all the networks, suggesting that these genera may serve as potential keystone taxa. The potential keystone taxa in the white spot portion fungal network based on peripherals are pathogenic genera such as *Exserohilum* and *Phaeosphaeria* based on while and *Bullera* and *Cladosporium* were identified based on connectors. In the spot proximity network, the genera *Exserohilum, Botrytis, Bipolaris,* and *Phaeosphaeria* were categorized into connectors, suggesting an increase in links with other modules (Table S4).

### 3.6. Functional Prediction of Microbial Communities

**Figure 8:**
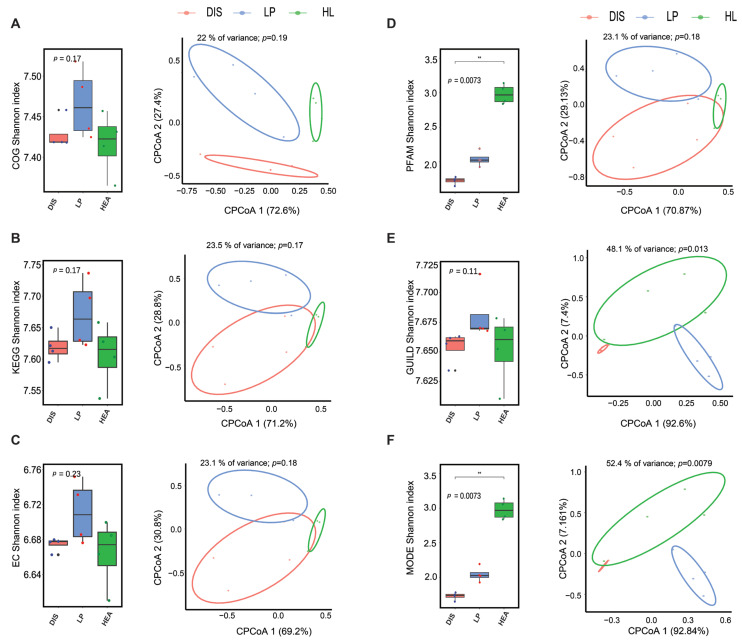
Alpha and beta diversities of microbial functional predictions in spot, lesion proximity, and healthy area of maize leaf. α-diversity and β-diversity functional profiles of bacterial communities based on the COG (**A**), KEGG (**B**), EC (**C**), PFAM (**D**), and fungal communities based on GUILD (**E**), and MODE (**F**). The asterisks represent the level of significance (** *p* < 0.01, Kruskal–Wallis test).

Picrust2 and FUNGuild databases were utilized to assess the potential functional profiles and changes in bacterial and fungal microbial communities in the maize white leaf spot portion, its proximity and healthy leaves. Functional genes were annotated using Cluster of Orthologous Groups of Proteins (COG), KEGG Orthology (KO), Enzyme Commission (EC), and (PFAM) databases for bacteria, while fungal functions were assigned based on Guild and Mode levels. The Shannon index and Constrained Principal Coordinate Analysis (CPCoA) were employed to visualize the functional differences between α and β-diversities levels (Figure 8). Analyses of functional α diversity showed a reduction in functionality in the diseased portion with significant differences in PFAM for bacteria and mode for fungi (*p* < 0.05) (Figure 8D,F). Constrained Principal Coordinate Analysis (CPCoA) of β-diversity functional profiles showed overlapping of all the samples in KEGG, EC, PFAM, fungal mode, and clear separation in COG and GUILD (Figure 8).

Furthermore, we investigated the abundance of genes in diseased, its proximity, and healthy samples. We found that genes such as thioredoxin system (Trx), methyl-accepting chemotaxis protein (mcp), ABC-2 type transport system ATP-binding protein (ABC-2.A), LacI family transcriptional regulator (lacI), and acyl-CoA thioester hydrolase (ybgC) were particularly abundant in the diseased sample compared to those in disease proximity and healthy samples. In contrast, genes involved in ion channel-forming bestrophin family protein (yneE), membrane fusion protein, multidrug efflux system (acrA), glutathione S-transferase (gst), and iron complex transport system permease protein (ABC.FEV.P) were more abundant in the healthy than in the diseased and its proximity samples (Figure S4A). 

The FUNGuild functional prediction of fungi showed that the characteristics of each sample varied with more abundant functional taxa in white spot proximity and healthy samples. In particular, the abundance of lichenized, plant–pathogen soil saprotroph, dung saprotroph, plant–pathogen, and plant–pathogen wood saprotroph were higher in the spot portion. Ectomycorrhizal, endophyte, saprotrophic, and fungal parasites were higher in healthy leaf samples. Surprisingly, a higher abundance of fungi with unassigned functions was observed in the diseased samples (Figure S4B).

## 4. Discussion

Plant microbiota comprises members that are useful, harmful, and neutral microorganisms [41]. Harmful or pathogenic microbes such as viruses, bacteria, and fungi cause disease and reduce the yield [42]. Even though plants have complex native immunity, such as pattern-triggered immunity and effector-triggered immunity, that help to protect and alleviate external stresses [43,44], plant-associated microbiomes also contribute to the resistance of harmful microbial invaders [45,46]. Despite the vital role of the phyllosphere microbiome in plant health, extensive research has primarily focused on the rhizosphere microbiome and its response to pathogens [47,48,49,50], with little attention given to the phyllosphere. In this study, we described the phyllosphere bacterial and fungal communities in the maize phyllosphere infected with maize white spot at both the infection site and its proximity. Our findings enhance our understanding of how the phyllosphere microbiome of the host plant responds to the pathogen infection in the infection site and its proximity.

### 4.1. Maize White Spot Infection Drives the Differentiation of Phyllosphere Microbial Diversity and Structures

By analyzing the microbial composition and diversity in the early and late stages of maize white spot development, we observed that the late stage of disease development and the proximity to white spot increased the alpha diversities of bacterial communities compared to healthy samples. In contrast, the alpha diversities of fungal communities decreased in the late stage of disease development. Specifically, our results indicated that the bacterial diversity was significantly higher in the diseased leaves compared to healthy ones. A significantly higher microbial diversity in plants may make plants less prone to pathogenic attack due to higher competition for resources [51,52].

A plethora of studies have demonstrated that infection by pathogens induces changes in the plant microbial communities [7,53]. Contrary to our findings, a previous study reported an increase in both bacterial and fungal alpha diversities in two healthy Malus species compared to the rust-infected samples [7]. Other studies have shown that the decrease in diversity and richness of the maize phyllosphere microbiome correlates with the increase in the severity of southern leaf blight disease and powdery mildew disease of pumpkin [54,55], which aligns with our findings in fungal communities (Figure 3B). The increase in bacterial community diversity due to maize white spot disease, particularly in the diseased sample, may be linked to bacterial responses and interactions with plant-native immune systems to mitigate pathogen infection [8].

Maize leaves infected with white spot, those in proximity to spot, and healthy leaves showed differences in bacterial and fungal community structures. Notably, the abundance of bacterial genus demonstrated that the abundance of the bacterial genus *Sphingomonas* was significantly enriched in diseased leaves, followed by those in proximity to white spot compared to healthy leaves. Plants are associated with both pathogenic and beneficial microorganisms. The pathogens employ various strategies to invade plant host immunity and establish disease, including the overriding of pattern-triggered immunity (PTI) and effector-triggered immunity (ETI) [8]. In response, plants recruit beneficial microbes to restrict the growth of pathogens [56]. These beneficial microbes can trigger the plant immune system and suppress pathogen invasion [57]. The mechanism of biocontrol in bacteria is mainly associated with antibiosis, competition for nutrients, and induction of plant host resistance [58]. A previous study demonstrated that *Sphingomonas* inhibits *Pseudomonas syringae* in the phyllosphere by disrupting pathogen chemotaxis and virulence [59]. The genus was also identified as an excellent inhibitor of *Diaporthe citri* growth [9]. Furthermore, the abundance of Bacteroides was considerably higher in the two samples compared with the healthy leaves. Bacteroides are pathogen-suppressing members of bacteria that play a role in host fitness [60,61]. Members of this genus enhance plant defense, induce systemic resistance, and produce plant hormones and siderophores [62]. This suggests that the increased abundance of these genera in the diseased and its proximity samples may be linked to protection against the maize white spot pathogen. Additionally, the genus *Chryseobacterium* was found in higher abundance in the spot portion and its proximity compared to the healthy sample. This genus has been reported to promote maize growth [63,64]**.**

The relative abundance of the potentially beneficial genera, *Stenotrophomonas,* and *Delftia,* were lower in disease compared to healthy leaf samples. *Delftia* has been reported to exhibit antagonistic activities against *Penicillium italicum* [65], while *Stenotrophomonas* spp. produce antimicrobial compounds, protect plants against infection, and promote plant growth [66]. Strains of *Stenotrophomonas* have been shown to interact with microorganisms in plant and soil surfaces through biofilms, inhibiting the growth of plant fungal and viral pathogens [67,68]. Furthermore, previous studies have also reported a reduction in the abundance of potentially beneficial bacterial genera taxa in plants such as *Sphingomonas* and *Bacillus* due to pathogen infections [69]. Enrichment of the plant’s potentially beneficial taxa was previously reported in the leaves of rice [8], cucumber [10], citrus [9], Malus [7], and wheat [70] after pathogens invasion.

The fungal community in the disease samples was dominated by the genus *Setophoma*. In contrast, the spot proximity and healthy leaves were dominated by potential pathogens such as *Symmetrospora, Cladosporium, Alternaria, Udeniomyces,* and *Exserohilum*. *Setophoma* spp. is a ubiquitous fungal pathogen that infects the roots of various plants, such as onion [71,72,73]. *Setophoma yingyisheniae* has been reported to cause leaf spot disease in tea plants in Yunnan [74]. The genera *Symmetrospora, Udeniomyces, Cladosporium, Alternaria, Exserohilium, Bullera,* and *Filobasidium* showed higher abundance in the lesion proximity and healthy leaf samples compared to diseased samples (Figure 6B and Figure S2). Some species belonging to *Cladosporium* have been reported to provide indirect protection ability against plant pathogens through enhancing resistance or directly by antibiosis and competition; they also protect plants against other abiotic stresses [75,76,77]. While some species of *Cladosporium* are known to be plant pathogens. For example, certain *Cladosporium* species were identified as causative agents of leaf spot of silage maize and leaf lesions on *Vicia faba* [78,79]. We speculate that the increase in the abundance of this genus in disease proximity, along with a drastic decrease in the diseased portion, may be associated with antimicrobial activity. A previous study reported an increase in beneficial bacteria such as *Pseudomonas* and *Bacillus* on the *G. yamadae* during initial infection and a drastic decrease at the later stage of infection [7]. *Exserohilum spp.* causes northern leaf blight of maize, a lethal foliar disease of maize [80], and some species like *Exserohilum rostratum* are known pathogen that causes maize leaf spot [81]. Surprisingly, the abundance of this pathogen was higher in the disease proximity and healthy leaves suggesting that these areas may be prone to infection.

### 4.2. Maize White Spot Decreases the Fungal Microbial Interactions

Our result of microbial community co-occurrence network interactions revealed that fungal communities were more sensitive to maize white spot disease than bacterial communities (Figure 6). Fewer nodes and connected networks were found in the disease sample and spot proximal area compared to healthy maize leaf fungal networks. Additionally, average degrees were lower in the diseased and its proximity than in the healthy fungal network. The decrease in the complexity of fungal networks due to pathogens in our study agrees with observations from previous studies [53]. Similarly, leaves of citrus-infected by the fungal pathogen *Diaporthe citri* showed a significant increase in the complexity of the microbial network [9]. The complexity of microbial interactions has been associated with an improved functioning ecosystem compared to less complex [82] and may also relate to stress mitigation [8]. In accordance with our finding, the reduction in network complexity has been reported with the expansion of rust infection on Malus leaves [7]. These findings suggest that maize white spot destabilizes the fungal interactions in both the disease portion and spot proximal area, rendering the leaves more prone to infection.

Microbial community interactions are essential for the identification of keystone microbes. The keystone taxa of the network were identified based on Zi and Pi using peripheral nodes (nodes have only a few links with other nodes within their modules) and connectors (nodes highly connected to several modules) [83]. Based on peripheral, maize white spot pathogenic genera *Phaeosphaeria* and *Exserohilum* were identified as keystone taxa in the white spot portion network, while *Bipolaris, Botrytis,* and *Exserohilum* were hub taxa in the spot proximal area network, acting as connectors. Peripheral nodes serve as specialists, while connector nodes play an important role in ecological processes [84]. Keystone taxa may directly or indirectly influence the microbial assembly as well as function acting as mediators between the plant and microbiome [41]. The identification of keystone taxa *Bipolaris, Botrytis,* and *Exserohilum* in the spot proximal area network suggests they may facilitate the expansion of the white spot pathogen in the maize leaves.

### 4.3. Maize White Spot Lessen the Abundance of Bacterial Functional Pathways

Based on bacterial functional prediction profiling, genes were found to be more abundant in healthy leaves than in diseased portion and its proximity microbiomes (Based on KO pathways) (Figure S4A). We identified genes such as thioredoxin system (Trx), methyl-accepting chemotaxis protein (mcp), ABC-2 type transport system ATP-binding protein (ABC-2.A), LacI family transcriptional regulator (lacI) and acyl-CoA thioester hydrolase (ybgC) that were particularly abundant in the diseased sample compared to diseased proximity and healthy leaves. Genes related to thioredoxin system (Trx) pathways enable plants to cope with environmental changes through metabolism and energy transduction [85]. Furthermore, members of the lacI transcriptional regulator regulate virulence gene expression and carbon metabolism in bacteria [86]. Putative ABC transport system ATP-binding protein (ABC.CD.A) functions as regulators and transporters of cellular processes generated by ATP [87]. The increase in the abundance of the bacteria related to these pathways in the diseased sample may be related to the response to the maize white spot pathogen. Microbial genes involved in glutathione S-transferase (gst), membrane fusion protein, multidrug efflux system (acrA), and iron complex transport system permease protein (ABC.FEV.P) were more abundant in the healthy leaf samples (Figure S4A). Glutathione S-transferase (gst) has been reported to play a role in bacterial protection against chemical and oxidative stresses [88,89]. Our finding aligns with a previous study that reported that Fusarium wilt could significantly decrease the microbial functional profiles of KO and COG in the upper stem epidermis in Chili [53]. These suggest that the functional characteristics of bacterial communities may change in response to pathogen infection sites.

## 5. Conclusions

Taken together, our study provides evidence of the response of maize phyllosphere microbiome to maize white spot based on the spot portion, spot proximal area, and healthy leaves. Our data suggested that microbial diversity was significantly affected in the spot portion and its proximity, indicated by an increase in bacterial diversity in the spot and its proximity samples, alongside a decrease in fungal diversity at the white spot site and its proximity. The infection site of the maize phyllosphere could recruit beneficial bacterial taxa that may protect against the pathogen, as revealed by the increase in abundance of *Sphingomonas, Chryseobacterium,* and *Bacteroides*. Surprisingly, both the white spot proximity and healthy leaves were enriched with maize white spot potential pathogenic fungi such as *Clasdosporium, Bullera, Alternaria,* and *Exserohilum.* Our findings emphasize that maize infected with white spot harbors more pathogenic fungi in the spot proximal area and the healthy areas. Thus, further study is needed to understand the role of these taxa in spot progression.

## Figures and Tables

**Table 1:** Microbial co-occurrence network topological characteristics.

Network Topology	Bacteria			Fungi		
	DIS	LP	HL	DIS	LP	HL
Number of nodes	43	43	44	18	80	102
Number of edges	50	55	49	18	169	270
Positive edges	17	24	23	10	76	114
Negative edges	33	31	26	8	93	156
Average degree	2.381	2.558	2.279	1.444	4.225	5.294
Clustering coefficient	0.021	0.165	0.056	0.024	0.045	0.085
Modularity	0.582	0.592	0.653	0.746	0.462	0.409
Average path length	4.48	3.581	4.54	1.821	3.208	2.984
Graph density	0.058	0.061	0.054	0.085	0.053	0.052

Note: DIS; Disease sample, LP; lesion proximity HL; healthy leave.

## Data Availability

The 16S rRNA and ITS sequencing data were deposited in NCBI SRA under the BioProject numbers PRJNA1149294.

## Supplementary Materials

The following supporting information can be downloaded at: https://www.mdpi.com/article/10.3390/biom15020252/s1, Figure S1: The box plots illustrate the significant differences among the top 10 bacterial genera across the three samples; Figure S2: The box plots illustrate the significant differences among the top 10 fungal genera across the three samples; Figure S3: The relative abundance of maize white spot potential fungal pathogenic genera in the spot portion, their proximal area, and health leaves; Figure S4: Functional prediction in diseased, lesion proximity, and healthy microbiome in maize leaf; Table S1: Effect of maize white spot location on bacterial community structure according to PERMANOVA; Table S2: Effect of maize white spot location on fungal community structure according to PERMANOVA; Table S3: Topological role of bacterial genera based on within and among-module connectivity; Table S4: Topological role of fungal genera based on within and among-module connectivity.
