# Supplementary material for: Microbiome Analysis of Area in Proximity to White Spot Lesions Reveals More Harmful Plant Pathogens in Maize
Source: Biomolecules. 2025 Feb 9;15(2):252. doi: 10.3390/biom15020252 (PMC11853329; doi:10.3390/biom15020252)
Supplement: Supplementary file 1 [file biomolecules-15-00252-s001.zip › biomolecules-3380379-2nd proof-suppl figure.pdf]

Microbiome Analysis of Area in Proximity to White Spot Lesions Reveals More Harmful Plant Pathogens in Maize

Sauban Musa Jibril [1,2], Yanping Hu [1,2], Kexin Yang [1,2], Jie Wu [1,2], Chengyun Li [1,2,*] and Yi Wang [1,2,*]

[1] State Key Laboratory for Conservation and Utilization of Bio-Resources in Yunnan, Yunnan Agricultural University, Kunming, China.

[2] Yunnan-CABI Joint Laboratory for Integrated Prevention and Control of Transboundary Pests, Yunnan Agricultural University, Kunming, Yunnan 650201, China.

*Correspondence: 2004165@ynau.edu.cn(C.L.); wyi_0114@ynau.edu.cn(Y.W.)

Supplementary figures.

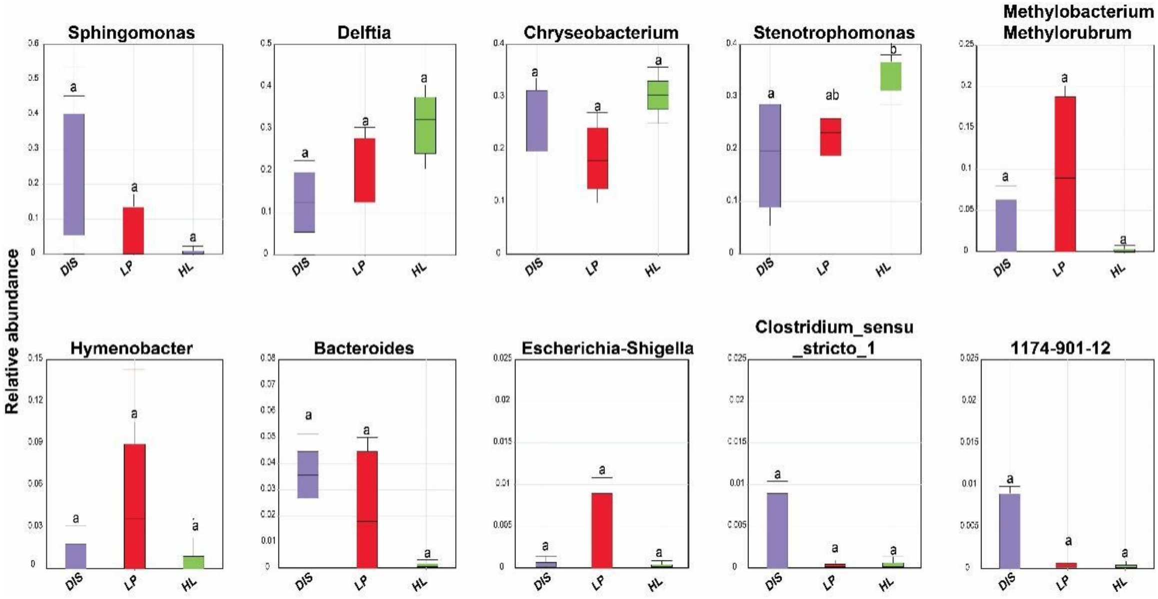

Figure S1. The box plots illustrate the significant differences among the top 10 bacterial genera across the three samples. The lowercase letters on each box plot display significant differences among samples (Duncan test, $p < 0.05$).

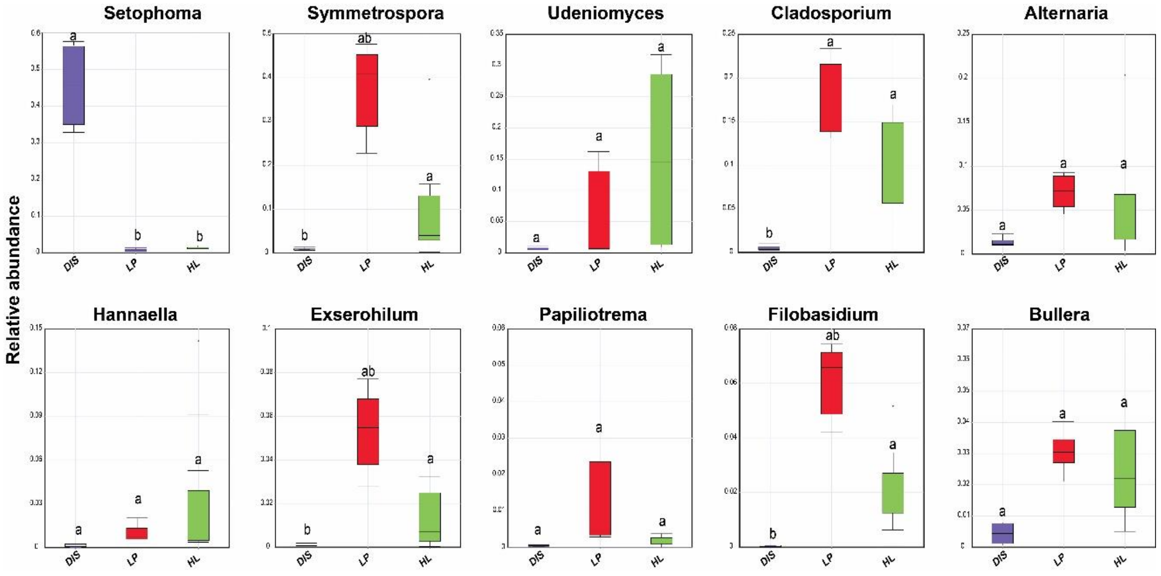

Figure S2. The box plots illustrate the significant differences among the top 10 fungal genera across the three samples. The lowercase letters on each box plot display significant differences between samples (Duncan test, $p < 0.05$).

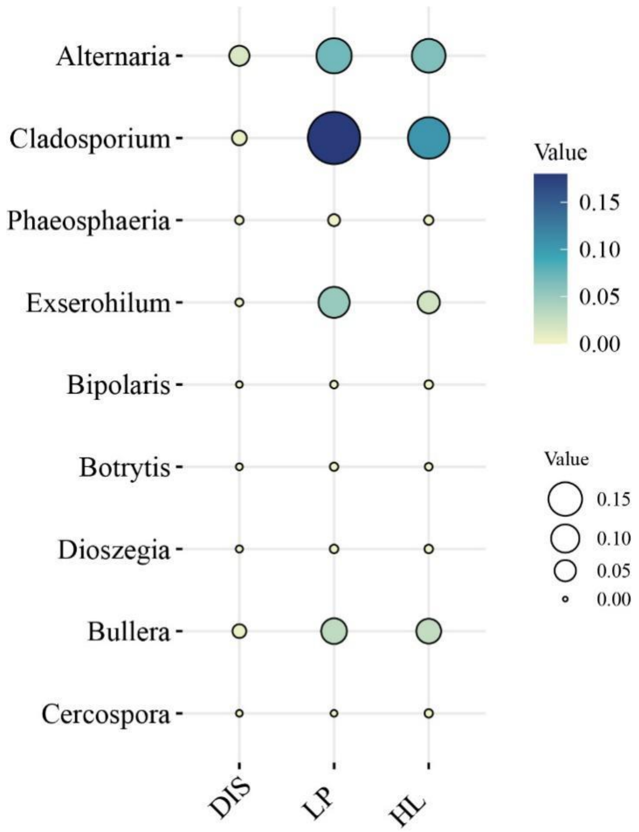

Figure S3. The relative abundance of maize white spot potential fungal pathogenic genera in the spot portion, spot proximal area and health leaves.

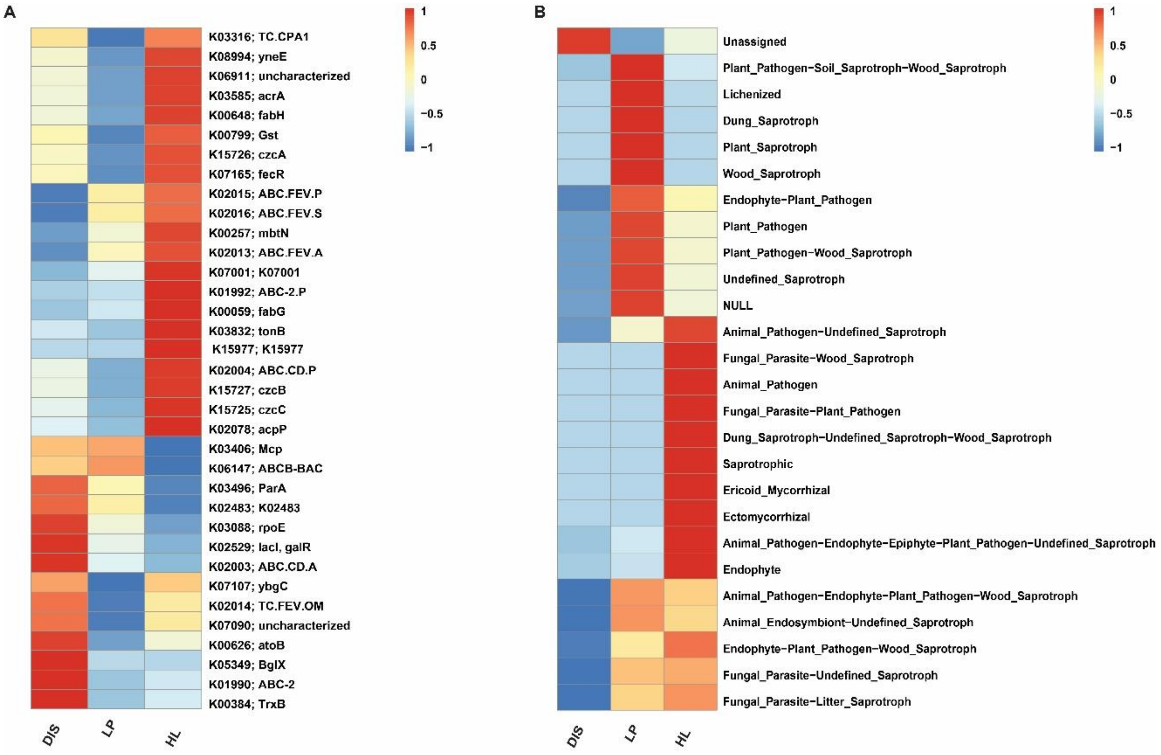

Figure S4. Functional prediction in diseased, lesion proximity, and healthy maize microbiome in bacterial **(A)** and fungal **(B)** communities.
